# Global longitudinal strain in aortic stenosis: risk versus remodeling

**DOI:** 10.1186/s44348-026-00101-9

**Published:** 2026-10-09

**Authors:** Inki Moon

**Affiliations:** Division of Cardiology, Department of Internal Medicine, Soonchunhyang University Bucheon Hospital, Bucheon, Korea

**Keywords:** Aortic valve stenosis, Global longitudinal strain, Ventricular remodeling, Aortic valve replacement, Echocardiography

In severe aortic stenosis (AS), left ventricular hypertrophy (LVH) begins as a compensatory response but can become a substrate for persistent risk. Concentric remodeling initially normalizes wall stress against a fixed obstruction, yet myocyte hypertrophy, interstitial matrix expansion, and replacement fibrosis may develop and persist even after the obstruction is relieved [1]. Aortic valve replacement (AVR) unloads the ventricle and initiates reverse remodeling, and the degree of LV mass index (LVMI) regression is prognostically meaningful: in the PARTNER trials and registries, each 10% reduction in LVMI at 1 year was associated with a 5% lower adjusted risk of subsequent all-cause death [2]. Whether that regression can be anticipated before intervention remains unresolved.

LV global longitudinal strain (GLS) is an attractive candidate, since it detects subendocardial dysfunction before LV ejection fraction (LVEF) declines and its prognostic value is well established: in an individual participant data meta-analysis of 1,067 asymptomatic patients with significant AS and preserved LVEF, impaired LV GLS was associated with a 2.62-fold higher risk of all-cause death [3]. Yet risk prediction is not equivalent to recovery prediction, and a ventricle at higher risk may still undergo substantial structural regression once afterload is removed.

In this issue of *Journal of Cardiovascular Imaging*, Purba et al. [4] examine this question in 94 patients with severe AS and preserved LVEF undergoing surgical AVR, using serial echocardiography at baseline, early after surgery, and at mid-term follow-up, with a complete-case cohort (*n*** = **54) as the principal analysis and baseline LVGLS evaluated both dichotomously, at a prespecified threshold of –16%, and continuously. Patients with impaired strain had more advanced hypertrophy at baseline (LVMI approximately 164 g/m^2^ vs. 111 g/m^2^), and the categorical analysis showed a significant LV GLS-by-time interaction for LVMI at follow-up (*P*** = **0.005), with greater mass regression in the impaired-strain group, but no interaction for septal thickness, relative wall thickness, or LVEF. As a continuous variable, LV GLS tracked absolute LVMI and septal thickness but not their change, and after multivariable adjustment it was not independently associated with any remodeling outcome.

Previous studies have not been consistent: in 83 patients with a design resembling the present study, Gelsomino et al. [5] identified baseline GLS as the strongest independent predictor of absent mass regression (odds ratio, 3.5; *P* < 0.001), whereas Kampaktsis et al. [6] found that baseline GLS did not predict remodeling after transcatheter AVR. Differences in endpoint definition, procedural approach, and follow-up duration may explain the discrepancy: the discriminating threshold in the study by Gelsomino et al. [5] was GLS ≥ –9.9%, well beyond the range separating groups here, and their follow-up extended to a median of 67 months, suggesting a nonlinear relationship apparent only at advanced dysfunction. The present findings argue against LV GLS as a broadly transportable predictor rather than proving it unrelated to every component of reverse remodeling.

The biology of reverse remodeling explains why a single functional measurement may fall short. Using extracellular volume mapping 1 year after AVR, Treibel et al. [7] found reductions of 22% in myocardial cell volume and 16% in matrix volume, while focal late gadolinium enhancement did not change. Cellular hypertrophy and diffuse matrix expansion can regress, whereas replacement fibrosis does not. LV GLS is an integrated, load-dependent signal shaped by contractile function and afterload, and one preoperative value cannot indicate how much of the hypertrophic burden is reversible. Tissue characterization is more specific: Ishiyama et al. [8] found greater LVMI reduction at 6 months in patients with lower preprocedural computed tomography–derived extracellular volume, despite comparable baseline mass and LVEF. Remodeling potential may therefore depend more on tissue composition than on longitudinal shortening alone.

Several limitations narrow the inference, beginning with a cohort that was small and unbalanced (22 vs. 72) and in which only 54 patients contributed mid-term data, leaving the principal analysis underpowered for modest effects. Date-based follow-up occurred approximately 8.5 months after surgery, whereas maximal mass regression required about 24 months in a series of 3,112 patients [9]. Although measured baseline and early postoperative variables were similar in patients with and without follow-up, 42.6% attrition still permits selection bias. Age and comorbidities were reported but not incorporated into the adjusted models, and New York Heart Association functional class was unavailable, so residual clinical confounding remains. No cardiovascular magnetic resonance was obtained, serial LV GLS was too incomplete to analyze, and clinical events were not captured.

An abnormal preoperative LV GLS should therefore not be interpreted as evidence that the myocardium is beyond structural benefit, and conversely, successful mass regression does not erase the residual risk that impaired strain signals. Reverse remodeling and clinical risk are related but distinct endpoints. This distinction also limits comparison with recent early intervention trials: the EARLY TAVR trial compared preemptive transcatheter AVR with surveillance in asymptomatic severe AS, whereas the EVOLVED trial tested a fibrosis-guided strategy; neither validated LV GLS as a stand-alone trigger for intervention [10, 11].

Measuring LV GLS in severe AS remains worthwhile, but the key question is how the number is used. It provides evidence of myocardial burden and may support closer surveillance and timely intervention within an integrated assessment, yet on present data it is not a reliable predictor of how much LVH will regress or whether AVR is worth performing. Impaired strain should heighten concern about residual risk, not diminish the expectation of benefit from relieving afterload.

## Data Availability

No datasets were generated or analysed during the current study.

## Abbreviations

AS: Aortic stenosis
AVR: Aortic valve replacement
GLS: Global longitudinal strain
LV: Left ventricular
LVEF: Left ventricular ejection fraction
LVH: Left ventricular hypertrophy
LVMI: Left ventricular mass index
